# Higher phagocytic activity of thioglycollate-elicited peritoneal macrophages is related to metabolic status of the cells

**DOI:** 10.1186/s12950-017-0151-x

**Published:** 2017-02-10

**Authors:** Sofia Pavlou, Luxi Wang, Heping Xu, Mei Chen

**Affiliations:** Centre for Experimental Medicine, School of Medicine, Dentistry & Biomedical Science, Queens University Belfast, Belfast, Northern Ireland UK

**Keywords:** Peritoneal macrophages, Phagocytosis, Function, Glycolysis, Extracellular acidification rate

## Abstract

**Background:**

Peritoneal macrophages are widely used in immunological studies. The cells can be collected under non-elicited (resident) or elicited (e.g., with Brewer thioglycollate broth injection) conditions, and their phenotype and functions differ. Recent studies have shown that macrophage phenotype and function are related to their metabolic states, and metabolic reprogramming has been an emerging concept for controlling macrophage function. In this study, we examined the metabolic state of resident and elicited macrophages and investigated how their metabolic state may affect cell function, including phagocytosis.

**Findings:**

Flow cytometry showed that elicited macrophages expressed higher levels of MHC-II, LFA-1 and CD64 but lower levels of F4/80 compared to naïve resident peritoneal macrophages, suggesting a more mature and active phenotype. Elicited macrophages had significantly higher levels of phagocytic activity compared to that of resident macrophages. Metabolic studies showed that the Extracellular Acidification Rates (ECAR) and Oxygen Consumption Rates (OCR) were both significantly higher in elicited macrophages than those in resident macrophages. The treatment of macrophages with 2-Deoxy-D-glucose suppressed glycolysis and reduced phagocytosis, whereas treatment with oligomycin enhanced glycolysis and increased phagocytosis in elicited macrophages.

**Conclusion:**

Naïve resident peritoneal macrophages are less metabolically active compared to elicited macrophages. Elicited macrophages had higher levels of glycolysis and oxidative phosphorylation, which may be related to their increased phagocytic capacity and higher levels of maturation and activation. Further understanding of the molecular links between metabolic pathways and cell function would be crucial to develop strategies to control macrophage function through metabolic reprogramming.

## Introduction

Macrophages are mononuclear cells distributed throughout the body and play a critical role in both innate and adaptive immunity. Peritoneal macrophages are useful tools to study the pathophysiology of tissue macrophages. The peritoneal lavage collection has been a well-established method of harvesting a relatively large number of fully differentiated macrophages from rodents [1]. Peritoneal macrophages are often collected either under non-elicited (resident macrophage) or elicited conditions (elicited macrophage), with sterile agents such as Brewer’s thioglycollate broth. The phenotypical and functional differences between resident and elicited peritoneal macrophages are well documented in the literature [2]. Recent evidence suggests that metabolic choices in cells enforce fate and function. How the phenotype and function of resident and elicited macrophages are regulated by metabolic pathways remains poorly defined. Therefore, in this study we investigated the metabolic state of resident and elicited macrophages and examined the link between metabolic state and function, in particular their phagocytic capacity.

## Methods

### Animals

12-week old C57BL/6 J mice were bred and maintained in the Biological Service Unit at Queens University Belfast in a 12 h-light/12 h-dark cycle with free access to water and chows. The use of animals was in compliance with the UK Home Office Animal (Scientific Procedures) Act 1986 and was approved by the local Ethical Review Committee.

### Cell collection

Peritoneal macrophages were harvested using the protocol as described previously [1]. Elicited macrophages were harvested from mice which had received a 1.5 ml intraperitoneal administration of 3% Brewer thioglycollate broth (Sigma, UK) 72 h prior collection. Resident macrophages were also harvested from non-injected mice.

### Flow cytometry

Cell suspensions were processed for staining of cell surface markers, including F4/80 (clone BM8), LFA-1 (clone 2D7), MHCII (clone AF6-120.1), CD16/CD32 (clone 2.4G2) (BD Biosciences, UK), CD64 (clone X54-5/7.1, BioLegend, UK). Data was collected using the BD FACSCanto II (BD Biosciences) and analysed using the FlowJo software (Tree Star Inc, CA, USA).

### Phagocytosis assays

Phagocytic activity of macrophages was assessed using the pHrodo *S.aureus* bioparticles conjugate phagocytosis kit and the Dextran-FITC phagocytosis system (ThermoFisher Scientific, UK) according to the manufacture’s instruction and previous publication [3]. Macrophages were seeded onto a black-walled 96-well plate for 2 h at a density of 5×10^4^/well followed by incubation with 2-Deoxy-D-glucose (2-DG) or oligomycin. The pHrodo *S.aureus* bioparticles were then added at a ratio of 10:1(bioparticles : macrophages) for 2 h before fluorescence intensity was measured using the Fluostar Omega plate reader (BMG Labtech). Culture medium alone with bioparticles was served as negative control. The net phagocytosis was calculated by subtracting the average fluorescence intensity of the no-cell negative controls from the experimental wells [3]. 1×10^5^ macrophages were incubated with Dextran-FITC (1 mg/ml) at 37 °C for 60 mins, and counter-stained with F4/80 antibody and analysed via FACSCanto II. Phagocytosis of dextran-FITC was expressed as MFI (mean fluorescent intensity).

### Cell viability

AlamarBlue® Cell Viability (ThermoFisher Scientific) was performed according to manufacturer’s guidelines.

### Bioenergetic study

Macrophage glycolytic function was assessed using Seahorse XF Glycolysis Stress Test Kit with a seahorse XF96 Analyser (all from Agilent Technologies, USA) following manufacturer’s instructions. Macrophages were seeded at a density of 3×10^6^ /well onto the 96-well microplate (Agilent Technologies) for 2 h prior to the assay. The glycolytic activities were assessed by measuring the Extracellular Acidification Rates (ECAR). Glycolysis is the ECAR after the addition of glucose. Glycolytic capacity is the increase in ECAR after the injection of oligomycin following glucose. Glycolytic reserve is the difference between glycolytic capacity and glycolysis. The Oxygen Consumption Rates (OCR) before any stimulation were calculated from the glycolysis stress test. Data were normalised with the cell number and expressed as mpH/min/10^3^ cells (ECAR) or pmol/min/10^3^ cells (OCR).

### Immunoblotting

Total protein extraction and Western blots were performed according to a previous publication [4]. Membranes were incubated with antibodies against glucose transporter 1 (Glut1, rabbit anti mouse, 1:1000, Millipore, UK), or β-actin (mouse anti-mouse, 1:2000, Santa Cruz Biotech, USA), followed by goat anti-rabbit IRDye® IgG 800CW or goat anti-mouse IRDye 680 conjugated secondary antibodies (LI-COR Biosciences, UK) respectively. Corresponding bands were detected using Odyssey infrared imaging system (Li-COR Biosciences). Densitometric analysis was performed using ImageJ software.

### Statistical analysis

Data were represented as mean ± SEM and analysed using unpaired, two-tailed Student’s *t*-test or one way ANOVA followed by Dunnett Test. *P <* 0.05 was considered statistically significant.

## Results and discussion

### Active Phagocytosis of elicited macrophages is associated with their phenotype

**Fig. 1:**
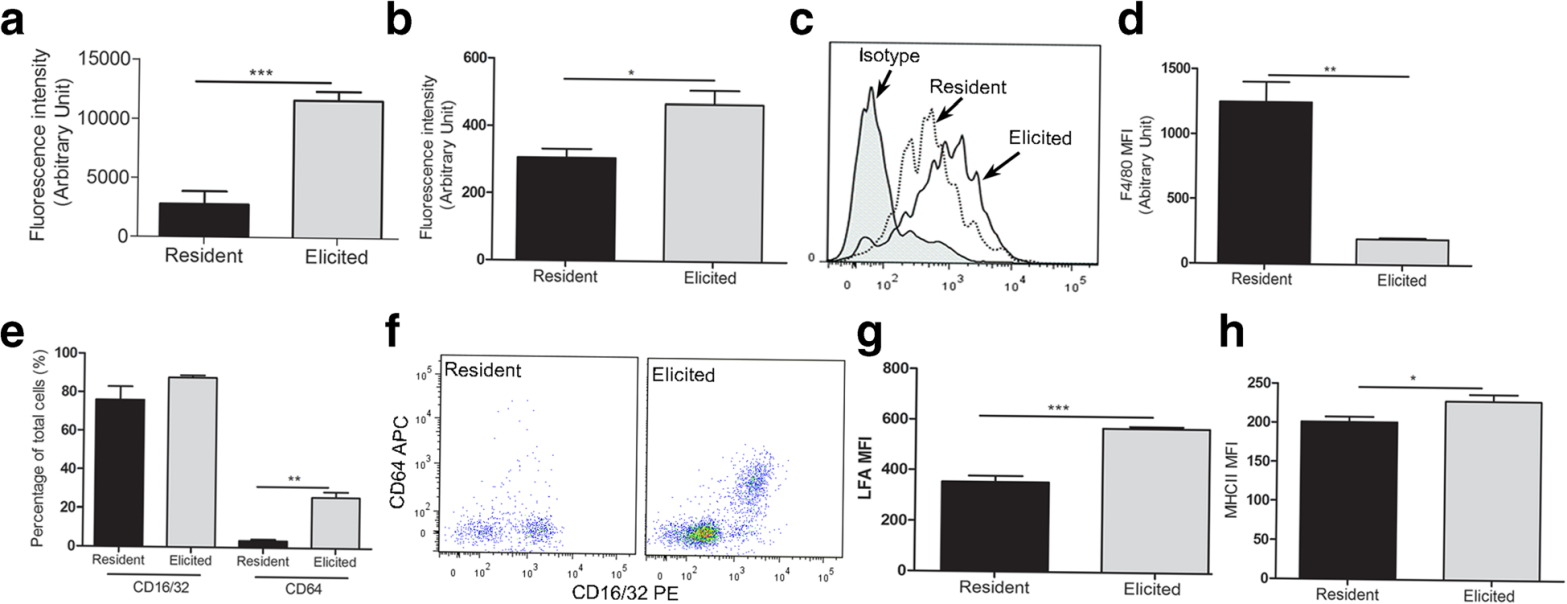
The phenotype and phagocytic function of resident and elicited peritoneal macrophages. Fresh peritoneal macrophages were processed for phagocytosis (**a**-**c**) or cell surface markers analysis (**d**-**h**). **a** Phagocytosis measured by the pHrodo *S.aureus* bioparticles assay. **b**, **c** Phagocytosis using the Dextran-FITC and measured by flow cytometry. **c** Representative histogram of Flow cytometry data showing Dextran-FITC uptake by macrophages. **d** Mean fluorescence intensity (MFI) of F4/80. **e** The percentage of CD16/CD32^+^ and CD64^+^ cells in resident and elicited peritoneal macrophages. **f** Representative dot-plot FACS images showing CD16/CD32 and CD64 expression in resident and elicited macrophages. **g** & **h** MFI of cell surface molecule LFA-1 (**g**) and MHC-II (**h**). Mean ± SEM, *N* = 4 mice. *p < 0.05; **p < 0.01; ***p < 0.001. Student’s *t* test

Phagocytosis is an inherent function of tissue macrophages and important for host protection and the initiation of the innate and adaptive immune response. Peritoneal macrophages are frequently used to study the phagocytic activity of tissue macrophages. The elicited macrophages demonstrated a significantly higher level of phagocytic activity compared to resident macrophages in both the pHrodo *S. aureus* bioparticles phagocytosis assay (Fig. 1a) and the Dextran-FITC phagocytosis assay (Fig. 1b-c). The pH based plate-reader method (Fig. 1a) revealed a higher level of difference between these two types of macrophages compared to the Dextran–based flow cytometric assay (Fig. 1b-c). Acidification is essential during phagosome maturation. The fluorescence of the pHrodo® dye dramatically increases as pH decreases from neutral to acidic in the phagolysosomes. This assay not only detects the uptake of particles, but also measures the formation of phagolysosomes. This data suggests that lysosomes in elicited macrophages are more active than those from resident macrophages.

To understand the mechanism underlying the difference in phagocytosis between resident and elicited macrophages, we further examined the phenotype of the cells. Flow cytometry analysis revealed higher fluorescence intensity of F4/80 in resident macrophages compared to elicited macrophages (Fig. 1d). The result is in line with a previous report in BALB/c mice [2], suggesting that the genetic background does not affect macrophage differentiation. Phagocytosis is often mediated by cell surface molecules such as scavenger receptors, mannose receptors, and complement receptors. Flow cytometric analysis revealed no significant difference on the percentage of CD16/CD32^+^ cells between resident and elicited macrophages (Fig. 1e). However the percentage of CD64^+^ cells was significantly lower in resident macrophages compared to that in elicited macrophages (Fig. 1e). Interestingly, almost all CD64^+^ macrophages expressed CD16/CD32 (Fig. 1f) on elicited macrophages. CD64 is recognised as a marker for mature tissue macrophages [5] and monocyte-derived dendritic cells [6]. Our data suggest that elicited macrophages are in a more mature state than resident macrophages. In addition, the elicited macrophages expressed higher levels of LFA-1 (Fig. 1g), and MHCII (Fig. 1h) compared to resident macrophages, suggesting higher levels of activation.

### Higher metabolic activity in elicited macrophages

**Fig. 2:**
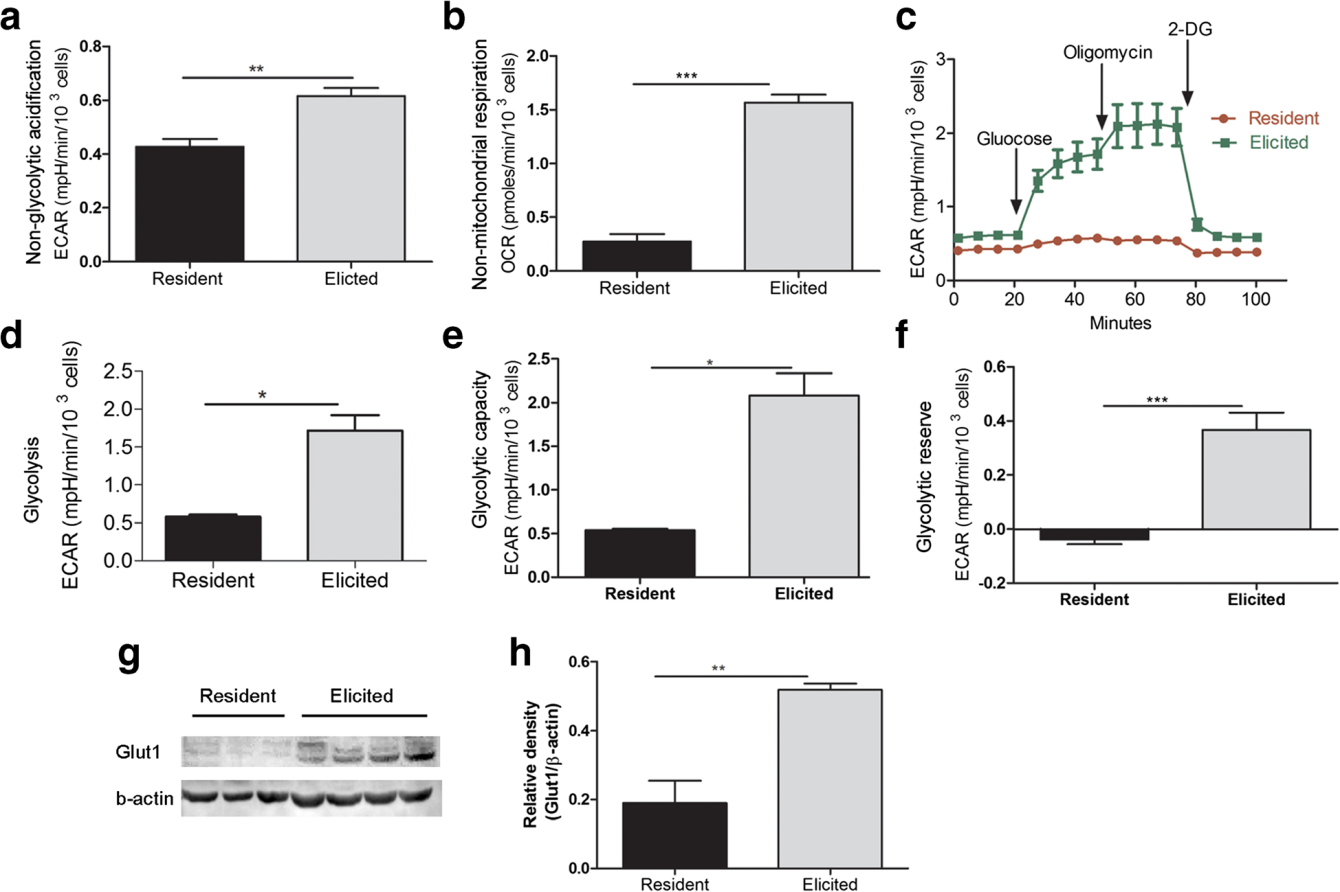
Glycolytic activities of elicited and resident macrophages. Fresh peritoneal macrophages were seeded in 96-well plate for 2 h and glycolytic activities were examined using XF Analyser. **a** Non-glycolytic acidification. **b** Non-mitochondrial respiration. **c** A representative graph output from XF96 showing ECAR response to glucose, oligomycin and 2-DG. **d** Glycolysis. **e** Glycolytic capacity. **f** Glycolytic reserve. (**g** & **h**) Western blot analysis of Glut 1. Mean ± SEM, *n* = 3-4 mice *p < 0.05, **P < 0.01, ***p < 0.001**. Student’s *t* test

Macrophage phagocytosis is fuelled by glycolysis and requires the presence of extracellular glucose [7, 8]. In the glucose-free, glutamine-present media, resident macrophages demonstrated lower levels of non-glycolytic acidification (Fig. 2a, *p* < 0.01) and non-mitochondrial respiration (Fig. 2b, *p* < 0.001) compared to elicited macrophages. Since these cells were equilibrated in glutamine containing media, we speculate that glutamine oxidation may contribute to the higher level of non-glycolytic acidification in elicited macrophages via the increased CO_2_ production in the mitochondria. These results suggest that resident macrophages utilise limited amounts of glucose and oxygen, and are metabolically inactive.

Using sequential addition of glucose, oligomycin and 2-DG in the XF96 analyzer, we further examined glycolysis, glycolytic reserve and glycolytic capacity of resident and elicited macrophages. After the addition of glucose, ECAR was significantly increased in elicited but not resident macrophages (Fig. 2c-d). When oligomycin was injected to inhibit oxidative phosphorylation, ECAR level was further increased in elicited macrophages, suggesting higher levels of glycolytic capacity (Fig. 2c, e). Resident macrophage failed to respond to the stimulation (Fig. 2c). Glycolytic reserve, the difference between glycolytic capacity and glycolysis rate was also significantly higher in elicited macrophages compared to that in resident macrophages (Fig. 2f). Our results suggest that elicited macrophages have a higher capacity to utilise glucose for energy supply and enforce functions.

Glut-1 is the major glucose transporter expressed in macrophages [9]. Western blot revealed a 2.75-fold increase in Glut-1 expression in elicited macrophages compared to resident macrophages (Fig. 2g-h, *p* < 0.01). Our results suggest that the elicited macrophages may uptake glucose through Glut-1 to maintain high metabolic activities.

#### Regulating macrophage phagocytic capacity by manipulating glycolysis

**Fig. 3:**
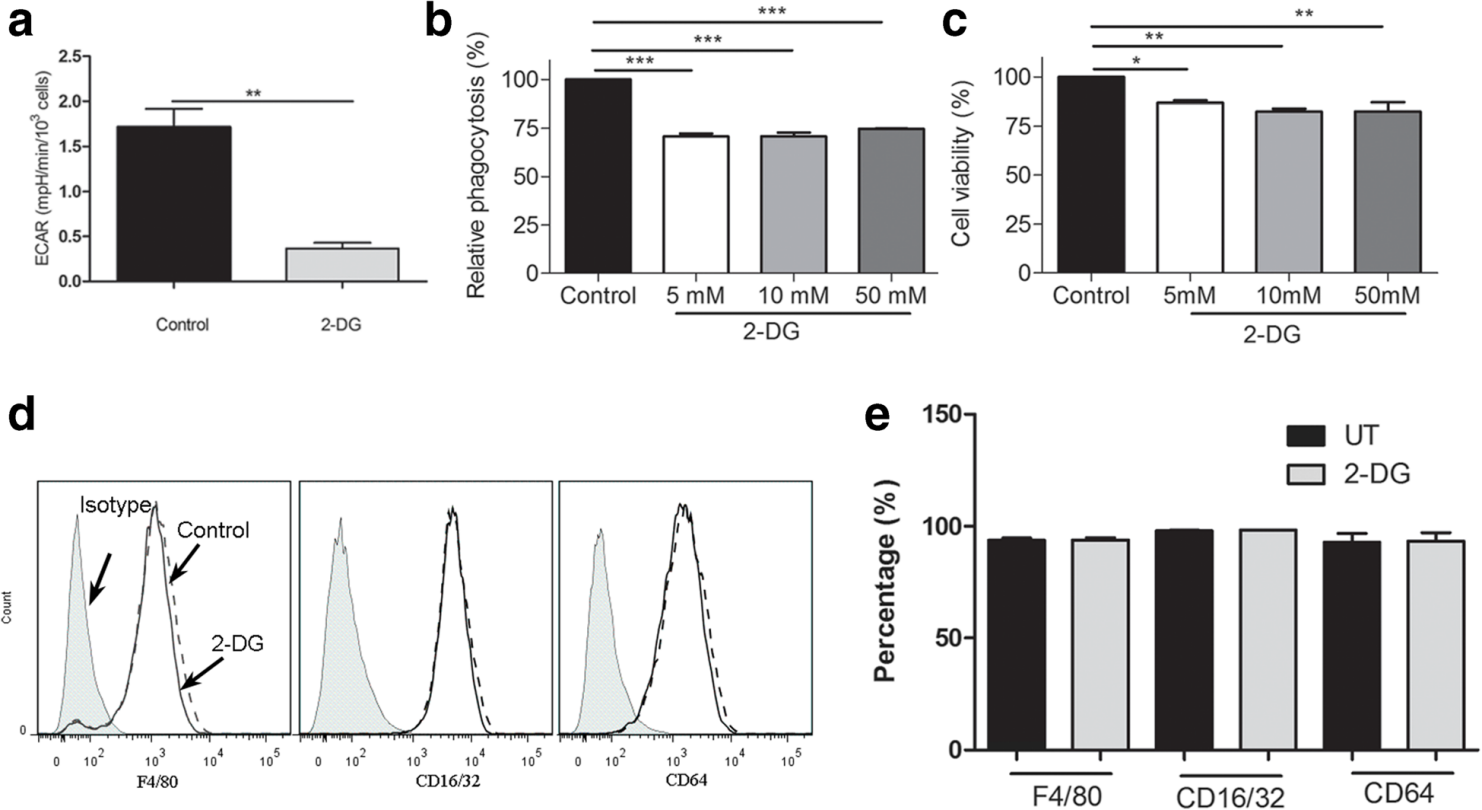
Effect of glycolysis inhibition in macrophage phagocytosis and phenotype. Fresh elicited peritoneal macrophages were treated with different concentrations of 2-DG for 1 h. Phagocytosis was measured using the pHrodo *S.aureus* bioparticles kit and cell viability by AlamarBlue® assay. The expression of F4/80, CD16/32, and CD64 was evaluated by flow cytometry. **a** ECAR in control and 2-DG treated macrophages. **b** Phagocytosis of pHrodo *S.aureus* bioparticles in control and 2-DG treated macrophages. **c** Macrophage viability following 2-DG treatment. **d** Representative flow cytometry data showing the expression levels of F4/80, CD16/32 and CD64 in control and 2-DG treated macrophages. **e** The percentage of F4/80^+^, CD16/32^+^ and CD64^+^ cells in control un-treated (UT) and 2-DG treated macrophages. Mean ± SEM, *N* = 4 mice. *p < 0.05, **p < 0.01, ***p < 0.001, One-way ANOVA, followed by Dunnett’s test

To further understand how macrophage phagocytosis is regulated by metabolic pathways, we used chemicals known to inhibit or enhance the glycolysis pathway. 2-DG, a nonmetabolizable glucose analog to glucose hexokinase inhibited glycolysis (Fig. 3a) and reduced phagocytosis (Fig. 3b) in macrophages, although the inhibitory effect was not dose-dependent. 2-DG treatment reduced cell viability (Fig. 3c). The expression of F4/80, CD16/CD32 or CD64 (Fig. 3d and e) in elicited macrophages remained unchanged, suggesting that the reduction in phagocytosis is likely due to the direct effect of glycolysis inhibition.

**Fig. 4:**
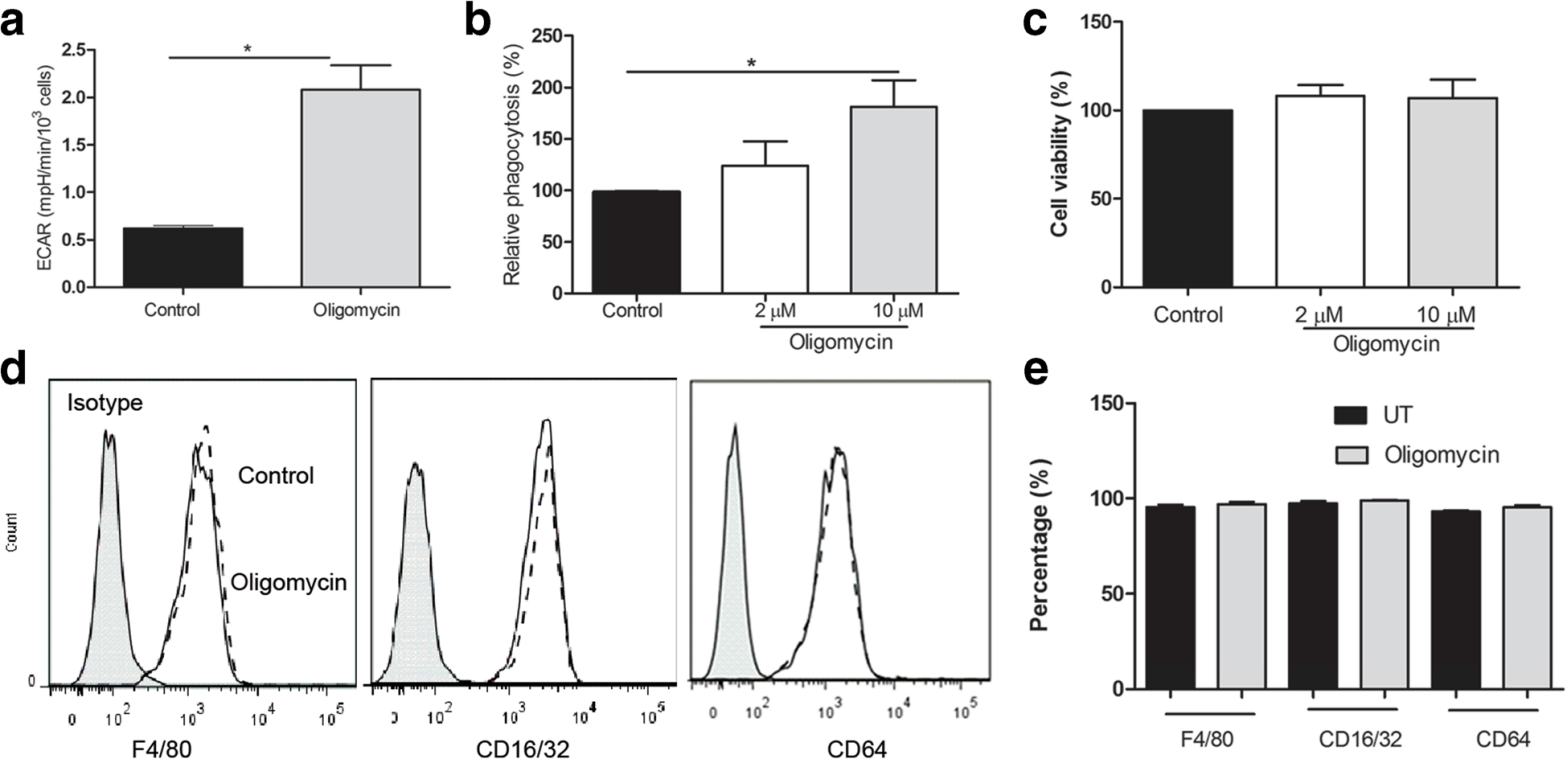
Effect of oligomycin in macrophage phagocytosis and phenotype. Fresh elicited peritoneal macrophages were treated with different concentrations of oligomycin for 15 min. Phagocytosis was measured using the pHrodo *S.aureus* bioparticles kit and cell viability by AlamarBlue® assay. The expression of F4/80, CD16/32, and CD64 was evaluated by flow cytometry. **a** ECAR in control and oligomycin treated cells. **b** Phagocytosis of pHrodo *S.aureus* bioparticles in control and oligomycin treated macrophages. **c** Macrophage viability following oligomycin treatment. **d** Representative flow cytometry data showing the expression levels of F4/80, CD16/32 and CD64 in control and olygomycin treated macrophages. **e** The percentage of F4/80^+^, CD16/32^+^ and CD64^+^ cells in un-treated (UT) and oligomycin treated macrophages. Mean ± SEM, *N* = 4 mice. *p < 0.05, Student *t* test in **a** and One-way ANOVA, followed by Dunnett’s test in **b**

Oligomycin inhibits mitochondrial oxidative phosphorylation by blocking the F0 subunit of the H^+^-ATPase. The treatment of macrophages with oligomycin increased glycolysis (Fig. 4a) and enhanced phagocytosis (Fig. 4b). Short-term (15 min) inhibition of mitochondrial oxidative phosphorylation did not affect cell viability (Fig. 4c) and the expression of F4/80, CD16/CD32 or CD64 (Fig. 3d and e) in elicited macrophages, suggesting that the enhanced phagocytic capacity is the direct result of metabolic switch.

## Conclusion

Elicited peritoneal macrophages are metabolically more active compared to naïve resident macrophages. Both the glycolysis and oxidative phosphorylation pathways are active in elicited macrophages. The metabolic difference may be related to the phenotypic and functional variations observed in these two types of macrophages, including the difference in phagocytosis. Further understanding the molecular mechanisms that control the metabolic pathways will shed light on the link between metabolism and function in macrophages.

## References

[1] Zhang X, Goncalves R, Mosser DM: The isolation and characterization of murine macrophages. Curr Protoc Immunol 2008, Chapter 14:Unit 14.1. 10.1002/0471142735.im1401s83 PMC2834554 19016445

[2] Turchyn LR, Baginski TJ, Renkiewicz RR, Lesch CA, Mobley JL (2007). Phenotypic and functional analysis of murine resident and induced peritoneal macrophages. Comp Med.

[3] Chen M, Hombrebueno JR, Luo C, Penalva R, Zhao J, Colhoun L, Pandi SP, Forrester JV, Xu H (2013). Age- and Light-Dependent Development of Localised Retinal Atrophy in CCL2(−/−)CX3CR1(GFP/GFP) Mice. PLoS One.

[4] Chen M, Lechner J, Zhao J, Toth L, Hogg R, Silvestri G, Kissenpfennig A, Chakravarthy U, Xu H (2016). STAT3 Activation in Circulating Monocytes Contributes to Neovascular Age-Related Macular Degeneration. Curr Mol Med.

[5] Gautier EL, Shay T, Miller J, Greter M, Jakubzick C, Ivanov S, Helft J, Chow A, Elpek KG, Gordonov S, Mazloom AR, Ma'ayan A, Chua WJ, Hansen TH, Turley SJ, Merad M, Randolph GJ (2012). Immunological Genome Consortium: Gene-expression profiles and transcriptional regulatory pathways that underlie the identity and diversity of mouse tissue macrophages. Nat Immunol.

[6] Langlet C, Tamoutounour S, Henri S, Luche H, Ardouin L, Gregoire C, Malissen B, Guilliams M (2012). CD64 expression distinguishes monocyte-derived and conventional dendritic cells and reveals their distinct role during intramuscular immunization. J Immunol.

[7] Venter G, Oerlemans FT, Wijers M, Willemse M, Fransen JA, Wieringa B (2014). Glucose controls morphodynamics of LPS-stimulated macrophages. PLoS One.

[8] Michl J, Ohlbaum DJ, Silverstein SC (1976). 2-Deoxyglucose selectively inhibits Fc and complement receptor-mediated phagocytosis in mouse peritoneal macrophages II. Dissociation of the inhibitory effects of 2-deoxyglucose on phagocytosis and ATP generation. J Exp Med.

[9] Freemerman AJ, Johnson AR, Sacks GN, Milner JJ, Kirk EL, Troester MA, Macintyre AN, Goraksha-Hicks P, Rathmell JC, Makowski L (2014). Metabolic reprogramming of macrophages: glucose transporter 1 (GLUT1)-mediated glucose metabolism drives a proinflammatory phenotype. J Biol Chem.

